# Petroleum sludge formation and its treatment methodologies: a review

**DOI:** 10.1007/s11356-023-31674-3

**Published:** 2024-01-04

**Authors:** Abdulraheim M. A. Hasan, Rasha S. Kamal, Reem K. Farag, Manar E. Abdel-raouf

**Affiliations:** Tanks Services Center (TSC), Egyptian Petroleum Research Institute (EPRI), 1 Ahmed Elzomor Street, Nasr City, Cairo, Egypt

**Keywords:** Residual tank deposits, Profitability decline, Physical treatment, Crude oil reclamation

## Abstract

Different petroleum operations produce huge amount of oil sludge annually. For instance, US EPA estimates the annual sludge production of each US refinery of 30,000 tons, while the average oily sludge produced from petrochemical industries in China is estimated about 3 million tons per year. In the last year, our center could recover about 30,206 barrels of raw oil from 32,786 barrels of tank bottom sludge (TBS) for different petroleum companies. This sludge causes huge economic losses besides its negative environmental impacts. The accumulation of sludge in the tanks results in reducing the tanks’ capacity for storing liquid crude, accelerating the corrosion of the tanks, delay in the production schedule, and disturbing the whole production operation. There are diverse treatment methodologies such as solvent treatment, addition of certain chemicals, and centrifuging. Of course, the environmental regulations and the overall cost limitations are very important in deciding the preferred applicable method(s). Although several works handled the problem of sludge deposition and treatment from different aspects, we intend to introduce a different work. First, composition, formation, types, and properties of TBS were reviewed. Then, environmental and economic problems caused by TBS were revised. At last, different methodologies applied for treatment of oily TBS to recover oil and safe disposal of hazardous remains were investigated focusing on the most straightforward and environmentally friendly protocols. It is expected that this review attracts the experts in petroleum chemistry, and other relevant fields and provides a comprehensive understanding of current sludge control and treatment research.

**Graphical abstract:**

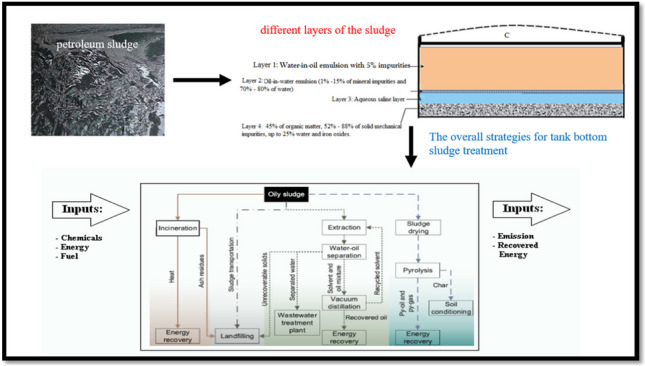

## Introduction

Petroleum crude oil is a fossil fuel that has been formed millions of years ago deeply under the ground. Typically, it is composed of different aliphatic, alicyclic, and polyaromatic hydrocarbons, some pyrene rings, minor elements, and heavy metals (Abdel-Raouf 2012a). The main components of crude oil are saturates (alkanes and cycloparaffins), aromatics (monoaromatics and polyaromatics), resins (polar molecules including heteroatoms such as S, O, and N), and asphaltenes (high-molecular-weight fused aromatic rings) which are examples of polar molecules (Abdel-Raouf et al. 2021; Abdel-Shafy and Mansour 2016; Mahmoud et al. 2021). This categorization is known as SARA test (Abdel-Raouf 2012a; Goual 2012). Of course, there are wide variation within this complex mixture between the oils produced from various oil reservoirs. Therefore, crude oils can be classified as paraffinic, naphthenic, waxy, or asphaltenic based on the proportions of various components within each category (Dahlmann and Kienhuis 2015; Goual 2012; Wong et al. 2015; Yang et al. 2022a; Yang et al. 2019; Zhang et al. 2018). Usually, metals including iron, zinc, copper, lead, molybdenum, cobalt, arsenic, manganese, chromium, vanadium, nickel, and sodium are found in wide concentration range from 1 to 1200 (Khuhawar et al. 2012; Silva et al. 2007; Vale et al. 2008). Indeed, the classification of oil according to its constituents serves many targets such as defining the type of the rock, estimating the amounts of different fractions that are attainable, and determining its physico-chemical properties important for transportation and processing (Peters and Moldowan 1993; Ramirez and Collins 2018; Simanzhenkov and Idem 2003).

Such information is very important because different products are obtained from each type. For instance, paraffinic oils with high content of short to medium carbon chain are fantastic for making aviation kerosene (jet fuel), diesel, lubricants, and paraffins. The manufacturing of petrol, solvents, and asphalt is best suited for aromatic oils, while naphthenic oils also contain notable amounts of petrol, naphtha, aviation fuel, and lubricants (Murungi and Sulaimon 2022). In fact, light crude oils refine into more gasoline, liquefied petroleum gases (LPG), and naphtha, giving them a higher marketable value, whereas heavy crude yields more liquid and solid components such as heavy paraffin oils and asphaltic materials (Abdulraheim M.A. Hasan, Mohamed Elkeshawy, Manar Abdel-raouf 2021). In general, the common derivatives, such as diesel and kerosene, are also crucial for road transportation. (Atta et al. 2004; Hasan and Abdel-Raouf 2018a).

**Fig. 1:**
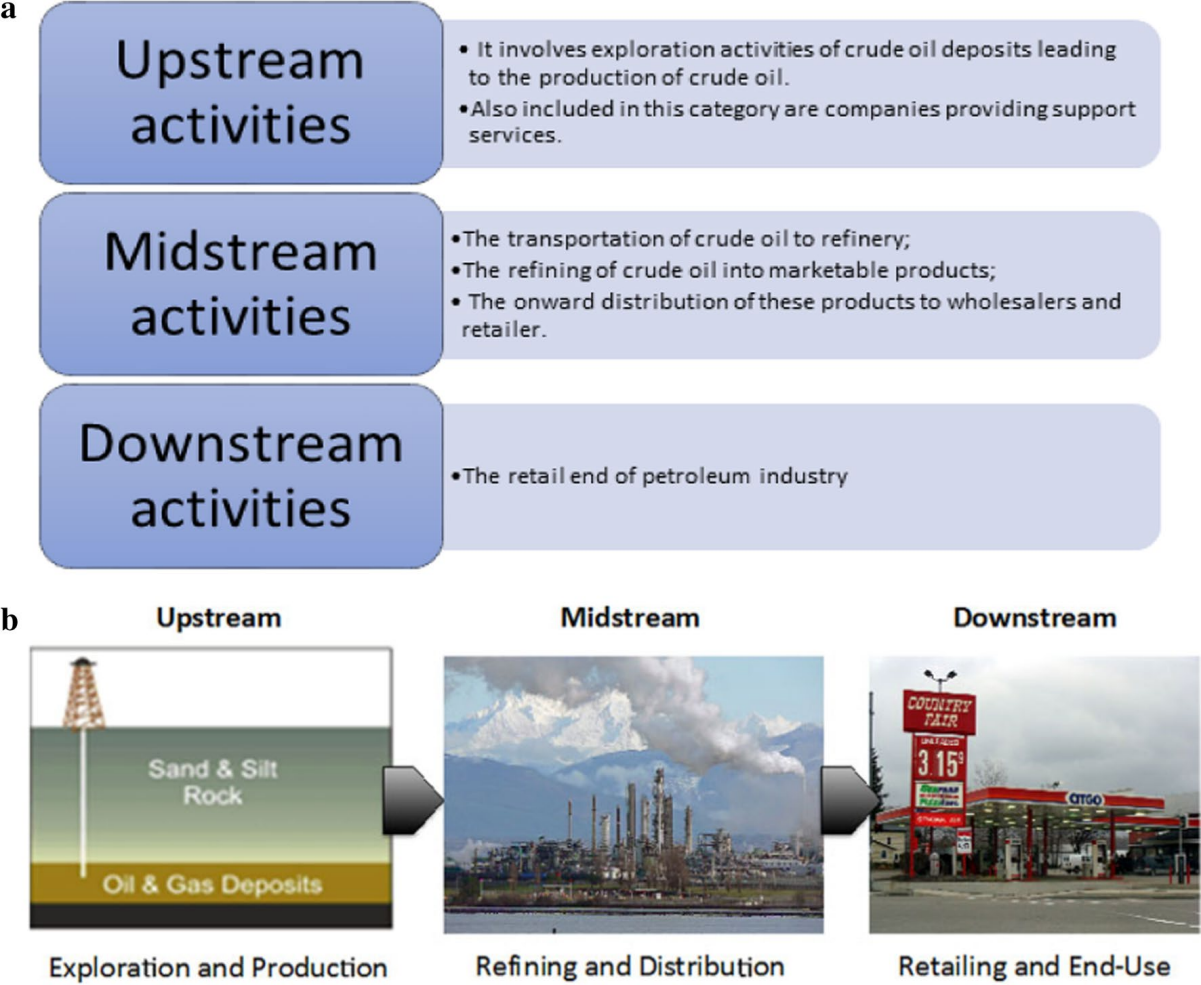
**a** The overall activities included in petroleum industry. **b** Chain of supply for petroleum-based products from well to consumer (*Compiled from* Wikipedia.org*) and* Wikimedia.org)

In spite of the great importance of petroleum crude oil as the main source of fossil fuels that have been used in all life aspects, it causes several economic and environmental problems during its overall processes (Abdel-Raouf 2012b; Elhaddad et al. 2015). Some of these problems are gathered in Table 1. Moreover, the processes applied to petroleum crude oil are illustrated in Figure 1a and the supply chain for petroleum goods from well to consumer is presented in Figure 1b.

**Table 1:** Some problems encountering petroleum industry during different processes

The process	The difficulties	Proposed solutions	Ref.
Production	The well rate, lift-gas rate, flow interaction, pipeline system deliverability, surface facility capacity for handling fluids, safety, and financial factors. Creation of water-in-crude-oil emulsions	Modifying lift-gas allocation rates, well rates of production, and production optimization, Demulsification	Pengju et al. (2002); Dhandhi et al. (2022)
Transportation	Corrosion of the pipelines Deposition of wax crystals Oil spills	Demulsification, dewaxing Addition of pour point depressants, physical and chemical methods of treatment	Abdel-Azim (2010); Abdul-Raheim et al. (2013a); Atta et al. (2013, 2006, 2004); Hart (2014); Iroha et al. (2021); Tripathy et al. (2021)
Refining	Gas flaring	The elimination of waste gases from the production stream and their subsequent flaring.	Sojinu and Ejeromedoghene (2019)
Storage	Tank bottom sludge	Different treatment methods	Azim et al. (2011); Emam (2015)

Although several works concerning resolving of the problems facing the petroleum industry have been conducted, we found few comprehensive works on the tank bottom sludge. Therefore, we introduce this work as an integrated review article on tank bottom sludge as one of the significant problematic issue that affects petroleum industry and sheds light on different strategies for treatment and demulsfication of carboneous residues accumulated in oil tanks including benefits and drawbacks for each technique. Finally, future construction projects will be handled to ensure they adhere to criteria for resource recycling and waste management.

Every year, various operations in the petroleum industry are thought to produce roughly 60 million tons of oil sludge (Hu et al. 2013). These sludges are thought to have produced the most significant amount of trash in the petroleum industry (Egazar’yants et al. 2015) (Shen et al. 2016). Additionally, it was estimated that sludges totaling more than a billion tons have been stored around the world (Mirghaffari 2017). It mostly interferes with the petroleum industry’s ability to operate and has a detrimental impact on the environment. Therefore, processing and demulsification of petroleum sludge are among the solutions of high economic value. Moreover, the extraction of oil from sludge can place the sludge among the petroleum-based fuels.

## Composition and properties of tank bottom sludge

Oil sludges are described by The Review of European List of Waste as “common hazardous wastes produced from petroleum refining, natural gas purification, and pyrolytic treatment of coal” and “oil wastes and wastes of liquid fuels” (Sander et al. 2008). Petroleum sludges are typically emulsions of the water-in-oil (W/O) type made up of hydrocarbons, water, and sediments (Hu et al. 2013). The inorganic minerals found in drilling fluids, storage tanks, discharges made while testing and maintaining wells, unintentional spills, and pipelines used in the oil sector make up the solid residue part of the sludges (Atta et al. 2006). Other minor components, including as metals, polyethers, and other compounds obtained during the refining of petroleum, may be present in the sludge (Phan et al. 2010). Some metals are introduced into sludge from oil additives. They are found as minor components, and the major metals found are lead (Pb), copper (Cu), nickel (Ni), vanadium (V), zinc (Zn), and chromium (Cr) (Kadiev et al. 2015).

Generally, the average composition of the petroleum sludge as proposed by some authors is given in Table 2.

**Table 2:** The proposed composition of the sludge

Water (%)	Sediments and undissolved materials (%)	Oil (%)	Ref.
30–90	3–7	5–60	da Silva et al. (2012)
30–50	10–12	30–50	Saikia et al. (2003)
30–70	2–15	30–90	Long et al. (2013b)
10–56	30–85	1–46	Egazar’yants et al. (2015)

This broad range shows that there is not even consensus among authors on the precise make-up of oil sludge. Briefly, the composition of each oil sludge is dependent on the composition and type of the crude oil it is generated from (Hu et al. 2013; Viana et al. 2015). For instance, Abdulqader et al. (2021) stated that the black oiy sludge obtained from Petronas in Melaka/ Malaysia composed of 78.91% moisture, 5.06% ash, 5.52% volatile matter and 10.51% solid carboneous materials. On the other hand, according to Alexandrovich and Sergeevich (2018), an oily sludge consists of organic components (72%), moisture (19.2%), sulfur (1.8%), and mineral portion (16%). The mineral composition of the prementioned oil mud was as follows: SiO_2_: 4.55%, CaO: 3.14%, Fe_2_O_3_: 1.65%, and Al_2_O_3_: 2.36%, and about 6% other metal oxides.

Therefore, the physicochemical features are dependent on the nature of the oil (Chen et al. 2020) and the creation and preservation of the oil sludge (Al-Doury 2019). The deposit amount is often lower than the oil and water contents (Hu et al. 2017). However, petroleum hydrocarbons are categorized in four major classes: aliphatics; aromatics; substances comprising nitrogen, sulphur, and oxygen (NSO); and asphaltenes (Hu et al. 2017). A full description of these classes is given in Table 3.

**Table 3:** Petroleum hydrocarbons existing in petroleum sludge

PHCs and heterocyclics	Average percentage	Constituents
Aliphatic hydrocarbons	75%	Alkanes, and cycloalkanes
Aromatic hydrocarbons		Naphthalene, phenols, polycyclic aromatic hydrocarbons (PAHs), such as phenanthrene, anthracene, chrysene, benzofluorene, and pyrene, as well as benzofluorene and its derivatives (toluene, xylenes), benzene, and its derivatives
Compounds that are nitrogen-sulfur-oxygen (NSO) containing	Less than 3% of the total composition is nitrogen (N). The amount of sulphur (S) is between 0.3 and 10%. Typically, the oxygen (O) level is less than 4.8%.	Naphthenic acids, mercaptans, thiophenes, and pyridines are examples of polar substances.
Asphaltenes		Mixtures of polyaromatic and alicyclic molecules with alkyl replacements, together with colloidal and pentane-insoluble compounds

From the chemical point of view, petroleum sludge typically contains 40–50% alkanes, 28–33% aromatics, 8–10% asphaltenes, and 7–22.4% resins by mass (Liu et al. 2019). Usually, the percentage of aliphatic hydrocarbon in crude oil is higher than the percentage aromatic hydrocarbon; about (40–60%) and (25–40%), respectively (Liu et al. 2019). As a result, many techniques and methodologies have been used to examine distinct forms of sludges (El-Naggar et al. 2011; Nezhdbahadori et al. 2018; Zubaidy and Abouelnasr 2010).

## Formation of petroleum sludge

Usually, the sludge derived from paraffin-rich petroleum is generated when the straight-chain hydrocarbons’ individual molecular orbitals are gathered together by closeness, resulted in an induced dipole force that enhance accumulation (Monteiro et al. 2014; Xu et al. 2018). Strong molecular combination is caused by these dipole forces, also known as London Dispersion Forces or Van der Waal linkages (Sidek et al. 2021).

**Fig. 2:**
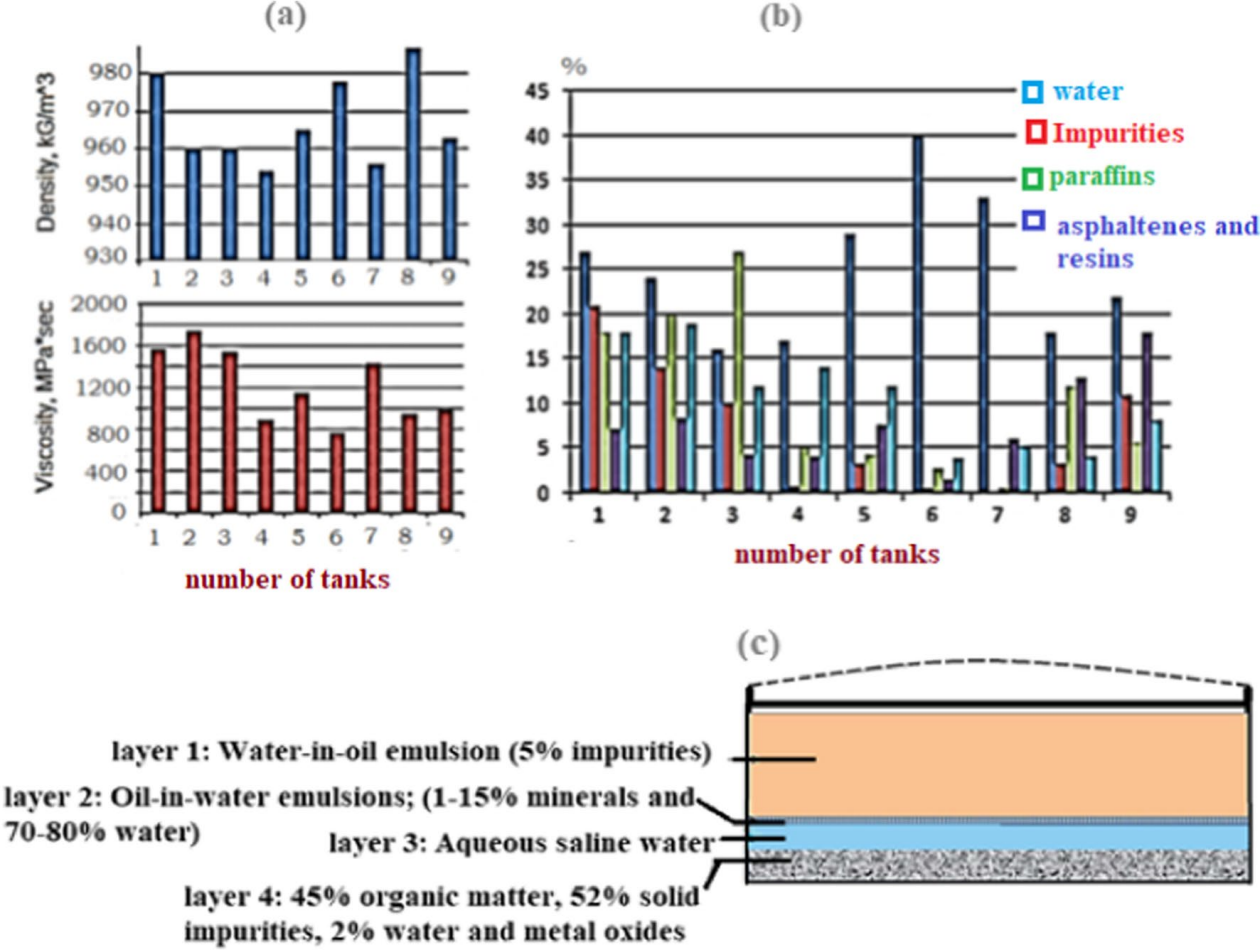
Parameters (**a**), crude oil sludge content (**b**) in various storage tanks, and (**c**) different sludge layers (Monteiro et al. 2015).

On the other hand, when the “heavier” straight-chain (C20+ hydrocarbon molecules) flocculate, they frequently phase out of suspension inside a static fluid, where they build up on the tank bottom as a dense, gelatinous substance. By time, with variations in temperature and pressure, the gel’s volatile components are “flashed” out, and the gel then thickens (Gao et al. 2018). It is also assumed that the sludge is formed when petroleum or petroleum products interact physiochemically with oxygen, moisture, and mechanical contaminants in a specific environment. This interaction results in different sludges with various compositions and physicochemical properties. Therefore, the sludge density may vary obviously. It has a pour point between −3 and +80 °C and a flash point between 35 and 120 °C (Egazar’yants et al. 2015; Heidarzadeh et al. 2010; Venkoparao and Sai 2013). This variation is illustrated in Fig. 2a where the chemistry of the sludge in these tanks is shown in Fig. 2b. along with the sludge density and viscosity for nine storage tanks. Generally, tank bottom sludge is formed in a multi-step process involving deposition of many layers, some of which might contain an emulsion of some kind (Monteiro et al. 2015). For instance, the upper layer of sludge is of type water-in oil emulsion with 5% or less of fine impurities. Asphaltenes, waxes, and resins, among other natural stabilizers, help to keep this emulsion stable. Then comes the middle, thin layer, which is an oil-in-water emulsion made up of between 70 and 80% water and roughly 1% to 15% of mineral species. The third layer that follows is made of supernatant salty water, which has a density of roughly 1.101 to 1.19 kg/m^3^. The bottom layer (sometimes referred to as “bottom sludge”) is the bottom layer and is the last layer. It is a solid phase that contains up to 45% organic matter, 52 to 88% solid mechanical impurities, up to 25% water, and iron oxides (Bahadori 2018). The sludge layers are displayed graphically in Fig. 2c.

## Types of tank bottom sludge

Generally, the type of the sludge is dependent on the of crude oil it comes from (Monteiro et al. 2015). However, naturally no two sludges are of the same composition; they are all highly dissimilar even if deposited from the same crude oil (Hu et al. 2013). This may be due to stepwise deposition of sludge layers. Based on the percentage of each component, the sludge can also be paraffinic, naphthenic, naphthenic-aromatic, and asphaltenic. On the other hand, there are main physicochemical constraints of tank bottom sludge that are crucial for judging its environmental impacts and giving technical information for deciding the appropriate handling protocols. They include-but not limited to- pH, EC, COD, TOC, the hydrocarbon fraction(s) composition, elemental composition, the concentration of trace elements such as heavy metals and metalloids, and non-metallic elements such as nitrogen and phosphorus (Bahadori 2018).

## Problems caused by petroleum deposits for petroleum sector

During different petroleum processes starting from exploration to refining, different slick deposits are generated (Monteiro et al. 2015). Crude sludges can be roughly categorized into the following groups according to where it came from:

The sludge produced in the upstream operations:

- It contains drilling mud remnants, crude oil tank bottom sediments, and fluids at oil wells (Hu et al. 2013).
- The sludge created in post-processing activities, such as solids, slop oil emulsion, and heat exchange bundle cleaning sludge, as well as leftovers from other systems, including:

- Oil-in-water separators, including CPI, parallel plate interceptors, and API separators.
- Sediments at the bottom of storage tanks, rail cars, and trucks.
- Sludge other units such as from FFU, DAF, or IAF units.
- Extra activated sludge from a local biological wastewater treatment facility.

However, the crude oil’s bottom sediments in storing tanks constitutes the majority of extensively concerned oily sludge in literatures due to their direct economic impacts on the total petroleum industry. This is normally because the separation of crude oil into heavier and lighter PHCs takes place in storage tanks before it is processed into petroleum-derived products. Once settled, the heavier PHCs frequently form an oily sludge combined with solid particles and water (Jiang et al. 2020). Those hard deposits are removed throughout tank cleaning procedures and transferred for further treatments or disposal (Kralova et al. 2011). The quantity and the volume of the sludge generated in the tank depend on multiple variables such as crude oil physical properties (e.g., density and viscosity), handling of refinery frameworks, oil storage method, and utmost importantly, the refining capacity (Kralova et al. 2011). It is worthy to mention that the total petroleum production rate in Egypt in this 2019 reached around 1.9 mboe/day of crude oil, natural gas, and condensates and LPG; this produced an average of 11,000 tons of oil sludge (Egypt’s Petroleum Sector: A Journey Of Success n.d.). Indeed, it was assessed that more than one billion tons of tank bottom sludges have been stored globally (Mirghaffari 2017). The US-EPA estimates that each refinery in the country generates 30,000 tons of oily sludge annually (EPA 1991). About three million tons of oily sludge from the petrochemical industry are produced annually in China (Wang et al. 2012).

In general, a greater refining capacity is usually linked to a higher amount of oily sludge production. Also, it has been assessed that every 500 tons of crude oil processed generates about one ton of oily sludge waste. This huge amount of the accumulated sludge highly affects the entire production process and hinders the refineries and causes remarkable economic loss. Additionally, this results in a decrease in storage capacity, poses risks to the oil tank’s functioning, and complicates maintenance and operation. The cost of sludge removal and disposal is part of the economic impact; the cost of disposing of the ecologically harmful waste is more expensive.

## Environmental impacts of oily and tank bottom sludges

As different types and quantities of oily sludge are formed under different processing operations, the environmental impacts of each type depend on several factors such as the location, timing, aging, and physicochemical features.

In this regard, Chen et al. (2018) studied the creation of polymer-containing oily wastes caused by the treatment of petroleum effluent in offshore oilfields. In contrast to oily sludge produced in oil storage tanks or during crude oil processing, oily sludge in oilfield waste open pits is stabilized through a strong interaction between the discarded oily wastes and the local environment, including solar radiation, atmospheric deposition, and subsurface soils.

Due to these environmental conditions, several processes can take place to the oily sludge such as evaporation of volatile short-chain petroleum hydrocarbons; evaporation and photocatalytic degradation of petroleum hydrocarbons also takes place by the effect of sunlight. The heavy fractions can then diffuse to the surroundings causing enormous soil and water pollution (Chen et al. 2018; Ul Haq et al. 2020). In their study on petroleum sludge from a flocculation/floatation unit in Sweden, Kriipsalu et al. (2008) found the polycyclic aromatic hydrocarbons (PAHs) naphthalene, fluorene, and phenanthrene predominated over the non-polar aliphatic hydrocarbons, which made up the majority of petroleum hydrocarbons. Different xylenes were the chief candidates in the BTEX group. All these compounds are very hazardous and proved to be potentially toxic candidates (Al-Mebayedh et al. 2022). In addition to short-chain petroleum hydrocarbons, oily sludge contains long-chain species like asphaltene and resin that are less evaporative and less degradable. These species are also enriched in some metals and cause an increase in the concentrations of heavy metal(loid)s that cannot be broken down. Moreover, the inorganic elements existing in petroleum sludge including some nuclides are all poisonous even at very low concentrations (Wang et al. 2017a).

In an interesting study, Wang et al. (2010) studied according to some characteristics, including life-time, growth, sheath output, generation production, and avoidance behavioral reaction as test endpoints, the toxicity of matured petroleum sludge to earthworm *Eisenia fetida*. They claimed that the harmful effects depended on concentration. In another work, Reinecke et al. (2016) assessed the ecotoxicity of soils in a farming area that include oily sludge. As a model organism, they used the earthworm *Eisenia andrei* (Oligochaeta). They discovered that low sludge concentrations had a negative impact on the biomass and reproduction of earthworms.

A prior field study was conducted by Hejazi et al. (2003) to verify the impact of oily sludge on potential human health risks at nearby land-farming locations in dry climates. They discovered that the vapors produced by oily sludge-borne hydrocarbons caused an astonishingly high concentration of airborne volatile organic compounds (VOCs) in the immediate vicinity, which could pose a serious risk to the workers on the site during the initial stages of the land farming process. Overall, oily sludge-borne species are potentially poisonous and could accumulate in plant tissues in addition to long- and short-term contamination of the air, soil, and water. Therefore, confinement, detoxification, and elimination are required as management methods for oily sludge, just like they are for other hazardous materials (API 1989).

Shortly, handling containment targets to restrict the spreading of oily sludge either keeping it in storage facilities or immobilizing it through solidification utilizing cementing ingredients, into the environment (Quan et al. 2022). Reclamation encompasses the elimination of hazardous substances in oily sludge to cut its risky effects via the physical separation or biological/chemical decay (Liu et al. 2021; Yao et al. 2010). Oily sludge’s organic components are meant to be entirely removed through high-temperature treatment in the elimination process (Gao et al. 2020). As most of these operative methods are very costly, there has been increasing attention focused on cost-effective alternatives for the use of oily sludge as it will be discussed in the next sections.

## Processing of oleaginous deposits

**Fig. 3:**
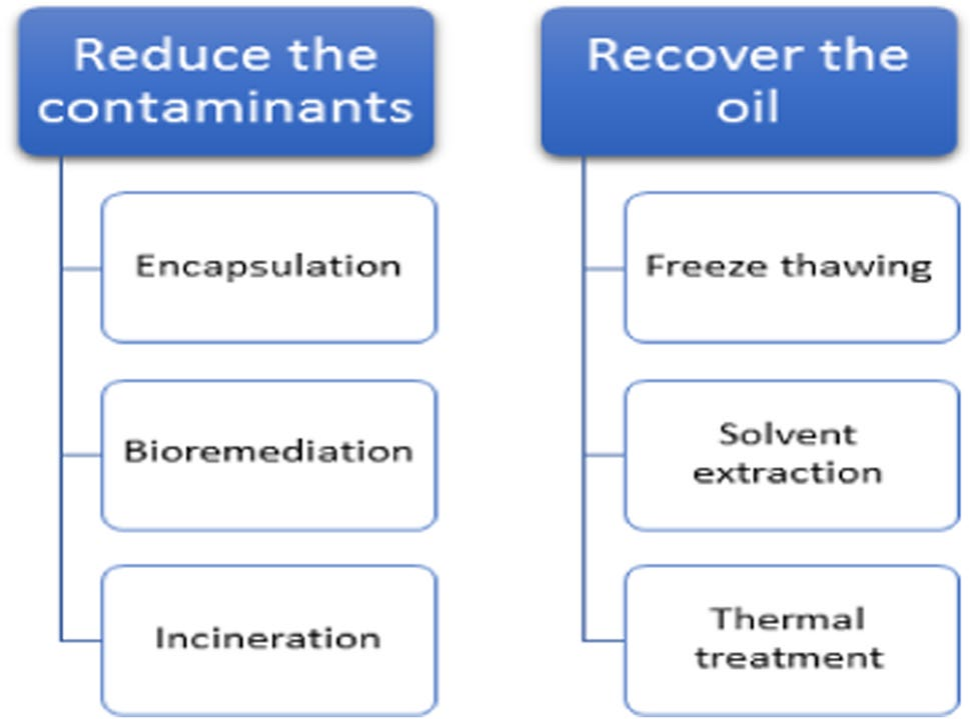
Treatment methodologies of tank bottom sludge: **a** oil recovery; **b** residue handling

Processing and analysis of petroleum sludge are very essential to make decision about the method(s) that will be applied for sludge treatment. Indeed, sludge composition is an important judging requirement to gain many benefits. These include recovering fuels and carbonaceous material residues from oily sludge, using of these deposits as feedstock for synthesis of composite materials, and extraction of metals and metal oxides. Moreover, the advance of cost-effective methodologies for the processing and valorization of oily sludge necessitates acquaintance of the physicochemical features of the chosen sludge to avoid its destructive impact on people and the environment. The composition and properties of oily sludge vary mainly according to its origin. Description and toxicity evaluation of oily sludge are therefore important in offering enough data required for effective handling oily sludge produced throughout different operations (Teng et al. 2021). Also, complete description of the sludge must be related to valorization, sustainability of the environment, and the circular economy. Initially, identifying different phases comprising the sludge can be conducted either physically (Jerez et al. 2021). or chemically (Ahmed and Nassar 2001). In this regard, Jerez et al. (2021) recognized the three phases of the oily sludge coming from an API separator (oily, aqueous, and solid phases) via fractional distillation process. The description of the sludge is discussed based on physical properties, and different methods have been suggested for the potential valorization of the oily sludge detailed in this work based on its chemical structure. Solvent extraction methodology was also applied by Zhao et al. (2020) to investigate the percentage of oil recovery under the effect of different solvents (n-heptane, cyclohexane, decalin, and ethylbenzene). On the other hand, Ramirez et al. used the NMR instrumental in the characterization of oil sludge from different sources (Ramirez et al. 2019). Their study is based on the extensive utilization of NMR in the identification of oil’s organic constituents (such aromatics, paraffins, and olefins) in sludges (Ma et al. 2019; Mazlova and Meshcheryakov 1999; Simpson et al. 2011). However, chemical demulsification of the petroleum sludge involves the addition of different surfactants and/or an oily solvent to repel out water and sediments from the sludge main body. In this regard, a successful three-phase separation was accomplished through the investigation conducted by Yao et al. (2022). They used different ratios of sodium lignosulfonate (SL)/sodium persulfate under many variables such as temperature, pH, and solid/liquid ratio. By this method, the oil recovery reached 89.65%. In another work conducted by Liang et al. (2017), the problem of residual solid particles has been solved by the addition of four types of surfactants into an oily sludge and the fate of solid particulates of petroleum wastes and the dispersion of oil and water partition coefficient and oil amputation efficiency have been followed up. The factors that govern the separation of oils from through chemical means oily muck include—but not limited to—the solid concentration, surfactant to solid ratio, sludge composition, and overall operating conditions. Generally, a collection of surfactant models was developed to account for all of these variables precisely. However, the processing of tank bottom sludge mainly aims to recover the oil and reduce the contaminants (Fig. 3).

### Treatment methodologies of tank bottom sludge

**Fig. 4:**
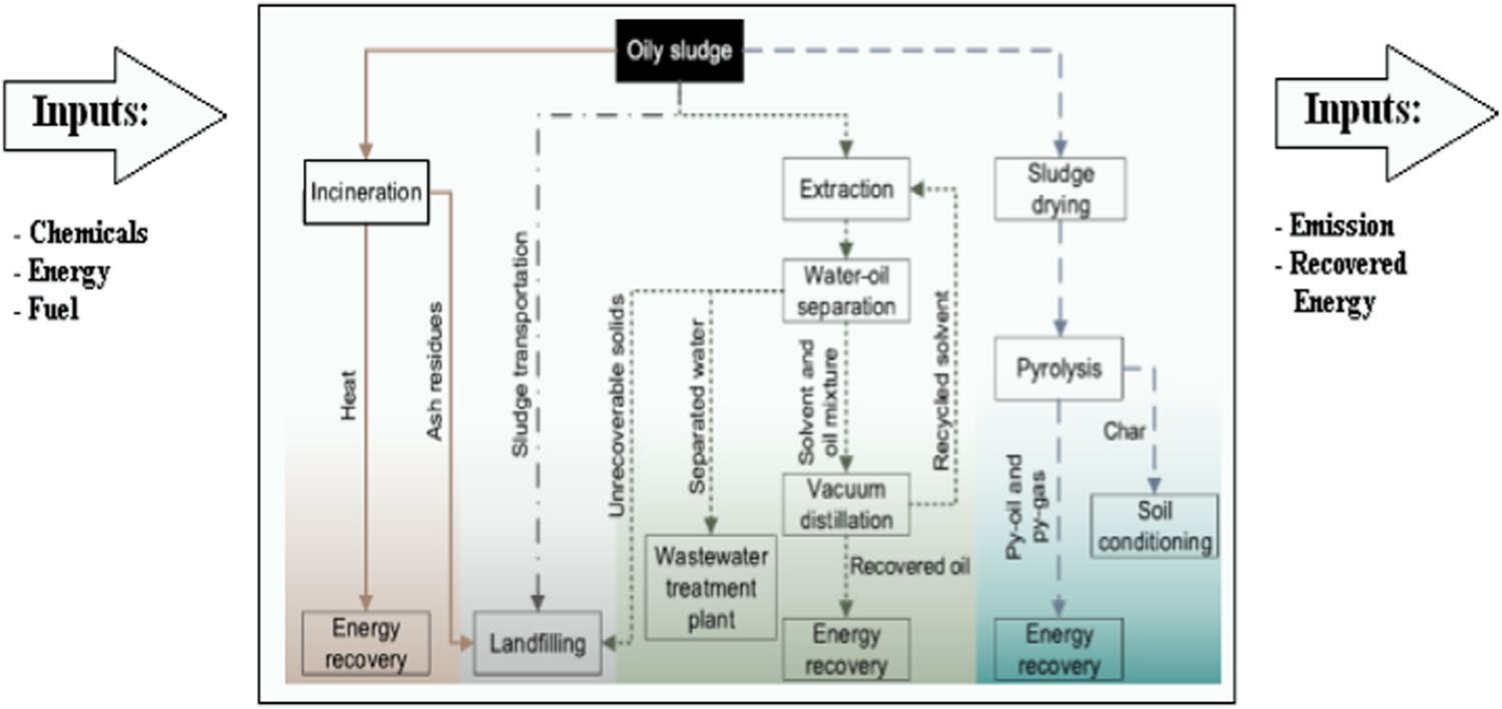
The overall strategies for tank bottom sludge treatment

Generally, the crucial strategy in petroleum sludge management relied on three pillars: (1) engaging technologies to reduce sludge generation throughout different stages in petroleum industry, (2) convalescing and retrieving valued fractions from present-day oily sludge, and (3) safe disposal of the remaining remains or if the first two phases don’t apply, the greasy sludge itself. However, once sludge is generated, the comprehensive management of oil sludges is aiming to either cut the contaminants in the sludge and/or to recover the oil, this classification is further illustrated in Fig. 3. Although the tank bottom sludge can be considered as a valuable source of fuel, the residue left after recovering the oil from the sludge must be carefully handled to reduce or eliminate the undesired pollutive effects it may induce. The overall strategies for oil sludge treatment are given in Fig. 4.

### Oil recovery

Valorization of petroleum sludge by recovery of oil and fuel is essential to offset the significant expenses and investments that are paid during different operations (Al-Futaisi et al. 2007). Therefore, different protocols have been developed in order to recover the maximum amounts of oils comprising the organic layer of the tank bottom sludge. Of course, the preferred ones are the cheapest, readily applicable, lower energy consumption, least processing facilities, and environmentally friendly (Hamidi et al. 2021; Hu et al. 2013; Liang et al. 2017; Sivagami et al. 2021; Teng et al. 2021). These methods will be focused and discussed within this review.

#### Solvent extraction

The process of solvent extraction is frequently used to dissolve volatile and non-volatile organic molecules from oil/water matrices. In this treatment, the oily wastes are dissolved with the defined single or mixed solvent(s) at the anticipated amounts to assure whole miscibility, despite the fact that water, solid particles, and hydrocarbon particulates are separated by solvent extraction. In order to separate the oil from the solvent, the solvent/oil mixture is next subjected to distillation. (Hu et al. 2013). In this context, many different solvents have been reported for treating oily sludge (Gazineu et al. 2005). Turpentine oil was used as a solvent to the oil removal, and it was discovered that the extracted oil accounted for 13–53% of the initial mass of the sludge. Zubaidy and Abouelnasr (2010) examined the results of a number of organic solvents, including MEK and LPGC under different variables such as the solvent/sludge ratio and temperature. The findings showed that MEK and LPGC at a solvent-to-sludge ratio of 4:1 had the highest oil recovery rates of 39% and 32%, respectively. In another study, El Naggar et al. (2010) employed various solvents for separating oil from both dry and semi-dry petroleum sludge, including naphtha cut, kerosene cut, n-heptane, toluene, methylene dichloride, ethylene dichloride, and diethyl ether, with toluene displaying the highest PHCs recovery percentage of 75.94%. Meyer et al. (2006) investigated the effect of several petroleum- based solvents on dissolving different components of tank bottom sludge. They found that petroleum-based solvent rich in ring compounds (such as aromatics and naphthenics) was extremely operative in dissolution in oily sludge, there are asphaltenic components, indicating that the recovery of sludge with more paraffinic (waxy) components was successful when using a solvent with a greater paraffinic character, like paraffinic diesel. Other organic solvents such as hydrocarbons from petroleum sludge have also been extracted using hexane and xylene as solvents; it was claimed that 67.5% of the PHCs in the sludge could be extracted, with the majority of them falling in the C9 to C25 range (Taiwo and Otolorin 2009). It is thought that high temperatures can accelerate the extraction process but also cause PHCs and solvent to evaporate, whereas low temperatures can diminish the effectiveness of oil recovery while saving money on the extraction process. (Fisher et al. 1997). Solvent thermo-degradation can be stopped by lowering the distillation temperature. The quantity and quality of recovered oil are also improved by the optimized solvent-to-sludge ratio. Fisher et al. (1997) stated that as the solvent concentrations increased, the amount of ash and high-molecular-weight hydrocarbons in the recovered oil dropped.

#### Chemical demulsfication of tank bottom sludge

**Fig. 5:**
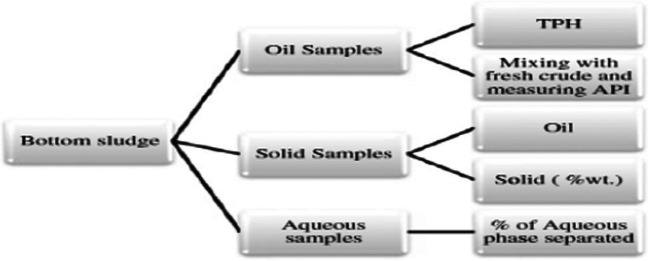
Analysis scheme of tank bottom sludge (Abdel Azim et al. 2011)

The method of breaking down petroleum sludge into oil phase, aqueous phase, and sediments is achieved either by solvent extraction or by addition of surfactants. Indeed, we have conducted several research papers concerning demulsification of petroleum sludge via addition of active ingredients. In our previous work (Abdel Azim et al. 2011), three nonyl phenol ethoxylate–based demulsifier systems (NP-9, NP-11, and NP-13) were introduced as cosurfactants in sludge breaker systems and applied on a real sludge sample obtained from the Al-Hamra Oil Company’s primary catchment area. Other constituents within these formulations include 4% inorganic acid solution, 10% of aqueous phase solution, and alcohols (isopropyl or butyl alcohol). A 1:1 combination of benzene and toluene make up the system’s remaining oil phase. The hydrocarbon phase was evaluated by TPH analysis and the gravimetric determination of phases separated from the sludge (aqueous phase and the sediments). The API of the combination was then computed after the oil phase retrieved from the sludge was blended in a 1:1 ratio with new crude oil acquired from Al-Hamra Oil Company. Additionally, the effect of the demulsifier formulation content and its concentration in parts per million was also explored. It was found that the optimum system that achieved maximum oil recovery was the one containing NP-13. The characterization strategies are displayed in Fig. 5. However, applying chemical surfactants can be accounted for emergent of toxicity to the environment and biological degradation resistance, among other issues. Thus, we have conducted some research articles concerning this issue. In order to eradicate the environmental pollution caused by the accumulation of plastic wastes, we employed poly (ethylene terephthalate) wastes for preparation of a series of ionic and non-ionic surfactants to be used as beach cleaners and benign breakers for petroleum sludge. The study disclosed that the prepared formulations showed high percentage of oil recovery exceeding 75% at very low concentration, about 200–250 ppm (Atta et al. 2004). In another work (Abdul-Raheim et al. 2013b), we introduced green, cost-effective sugar-based demulsifiers for petroleum sludge. In this work, 12 ethoxylated glucose esters were prepared by a green methodology and employed as potent demulsifiers for sludge samples received from Petrobel company for Belaeim field. The analysis scheme applied throughout this work is given in Fig. 4. In addition, we have developed different green surfactants as demulsifiers for different types of crudes (Abdel-Raouf et al. 2011a; Azim et al. 2010). In future works, these demulsifiers can be verified on soft and hard sludge.

Chemical treatment was used to examine oil recovery from oily sludge produced by the petroleum industry. To separate the oil phase from the oily sludge in this manner, three surfactants—cationic cetyltrimethylammonium bromide (CTAB), nonionic Tween 60, and anionic sodium dodecyl benzenesulfonate (SDBS)—along with an alkali, NaOH, were used. Basically, the addition of chemical agents into the sludge may involve mechanical stirring in order to distribute the additive efficiently (Liang et al. 2017). In some cases, addition of surfactants followed by thermal treatment enhances the deoiling process and increases percentage of oil recovery. It is assumed that increased temperature helps in film thinning and promotes film rupture which finally lead to an enhanced phase separation (Abdel-Raouf et al. 2011b; Al-Sabagh et al. 2004; Hasan et al. 2021; Hasan and Abdel-Raouf 2018b).

On the other hand, bio-surfactants find great importance to overcome the hazardous effects of synthesized surfactants to the surrounding environments. They are biologically borne (yeast or bacteria) from various nutritive media like sugars, oils, alkanes, and wastes. Five categories have been established. Glycolipids, lipopeptides, phospholipids, fatty acids, and neutral lipids, polymetric bio-surfactant, and particulate bio-surfactant are just a few of the substances that make up lipids. (Hasan and Abdel-Raouf 2018a). The majority of the bio-surfactants are either anionic or neutral, and the cationic ones are those containing amine groups. Among diverse biosurfactants, three groups have been broadly investigate. These include surfactins (a form of Lipopeptide) generated by Bacillus subtilis, rhamnolipids (i.e., a type of glycolipid) produced by Pseudomonas aeruginosa, and sorphorolipids (i.e., a type of glycolipid) produced by Candida bombicola. (Lai et al. 2009; Lima et al. 2011). Long et al. (2013a) reported that a bio-surfactant called rhamnolipid dewatered floating greasy sludge.

Suganthi et al. (2018) introduced a boost in the rate of enhanced biodegradation of hydrocarbons in the tank bottom oil sludge by maintaining a steady concentration of biosurfactants and enzymes including oxidoreductase, catalase, and lipase. The removal of heavy metals under the influence of specific bacterial species was also discussed. The biodegradation of oil sludge was accomplished using the microbial consortium *Shewanalla chilikensis*, *Bacillus firmus*, and *Halomonas hamiltonii*. The bacterial connections continuously produced lipoprotein biosurfactant (152 mg/g of oil sludge) and degraded the oil sludge by synthesizing several biocatalysts, including lipase (80 U/ml), catalase (46 U/ml), and oxidoreductase (68 U/ml). This resulted in a 96% decrease in the total petroleum hydrocarbons.

#### Thermal treatment of tank bottom sludge

The process of thermal treatment is usually followed by or combined with a pretreatment process either chemically by the addition of demulsifiers or physically by solvent treatment. Generally, thermal treatment refers to heating up the pre-treated sludge as a green and cost-effective approach. Although hot steam is commonly used in the production of petroleum, particularly for heavy and thick oil resources, it is rarely used to extract oil from oily sludge. Xia et al. (2020) developed a practical and reliable way for steam-assisted flotation treatment to recover the top oil layer. In essence, concentrated steam between 200 and 350 °C with a steam to oily sludge ratio (S/OS) of 2 to 8 was injected into the surface layer. 300 °C and a S/OS ratio of 6:1 were the ideal application conditions for optimal oil recovery. Within 5 min, 90% of the surface oil was collected, and it was subsequently dewatered for simpler transportation. They used the impact of high-temperature steam on the viscosity of the surface emulsion and the movement of the oil to explain their data.

Gupta et al. could recover about 90% of valuable hydrocarbon cuts from the sludge accumulated at the bottom of the tanks holding crude oil (Gupta et al. 2021). They applied a protocol involving simultaneous zeolite hydrocarbon recovery, catalyzed sludge cracking, and methane elimination. At 500 °C and 1 atm pressure, investigations were carried out with N_2_ or H_2_ as the carrier gas and two kinds of parent and desilicated zeolites (ZSM-5 and zeolite Y). Based to the carbon number range that corresponds to petrol, kerosene, and gas oil (heavy oil), paraffin wax and bitumen), the products were categorized.

In another work conducted by Wang et al. (2017b), ZSM-5 zeolites were applied as catalysts to endorse the aromatization during pyrolysis of oily sludge. The overall aromatic yield and product distribution were used to gauge the effectiveness of the catalysts and the impact of the operating conditions. Results showed that ZSM-5-O, with a silicon/aluminum ratio (SAR) of 19, was capable of producing the highest catalytic activity (approximately 93.37%) at a retention period of around 40 s. Longer retention times and larger catalyst dosages resulted in an overall improvement in the yield of aromatic. The reaction-regeneration cycles improved the tricyclic aromatic hydrocarbon content, and the renewed zeolites shown equal catalytic activity for aromatization. However, pyrolysis of tank bottom sludge was established as an effective methodology for oil recovery. Generally, the term pyrolysis refers to organic compounds undergo thermal breakdown at high temperatures (500–1000 °C) in an inert environment. Lower molecular weight hydrocarbons in liquid condensation or non-condensable gases are the end products of the pyrolysis process. Additionally, it produces char, a solid byproduct (Zango 2020) . The proportions of these products are mainly dependent on the operational conditions (Zango 2020). Also, the main products of pyrolysis are mixture of different phases such as char, liquid, or gas, and they may have higher calorific values than the raw oily sludge (Butler et al. 2011).

Several experimental works have been conducted for investigation the effect of pyrolysis on fuel recovery from oily sludge. Shen and Zhang (2003) noticed that oil yield essentially increased linearly with pyrolysis temperature, reaching its peak at 525 °C (30 weight percent of the feed oily sludge), but abruptly decreased above that temperature due to secondary decomposition reactions and the oil’s breakdown into lighter and gaseous hydrocarbons.

In another study, Liu et al. (2009) found that about 80% of stable hydrocarbons could be recovered at pyrolysis temperature range of 327–450 °C. Beside recovery of different fuels from the sludge, the char produced from pyrolysis can be used as a source of fuels in power plants (Wang et al. 2018). Other pyrolysis processes and the products resulted from each process are tabulated in Table 4.

**Table 4:** Products of pyrolysis of different tank bottom sludges

The pyrolysis conditions	The yield	Ref.
Fluidized bed and temperature range of 460–650 °C	70–84% of oil	Deng et al. (2015)
Co-pyrolysis of rice husk and petroleum sludge at 600 °C in a fixed bed reactor	While resin and asphaltene concentration decreased by 25–31% and 11–68%, respectively, saturates and aromatics in oil products increased by 15–55% and 55–86%, significantly.	Lin et al. (2018)
The interaction between accompanying the heat of reaction throughout the drying and combustion processes of oily sludge, the properties of combustion of oily sludge with fuel	Mixing biomass with oily sludge could enhance the incineration of oily sludge. At various temperatures, distinct interactions between the burning of oily sludge and biomass were found. The interaction reduced the combustion of the mixes between 280 and 390 °C, while it accelerated it between 390 and 620 °C.	Deng et al. (2016)
Using combined thermal cracking and catalytic pyrolysis to recover oil from oily sludge	The oil output improved as the temperature rose over 450 °C and peaked at 750 °C (76.84 wt%).	Jin et al. (2021)
Microwave pyrolysis oil recovery	Highest oil yield was obtained at the following optimized conditions: 450 W power using a 1:5 ratio of graphite to sludge. Oil and char were determined to have calorific values of 44,442.9 and 16,686.58 kJ/kg, respectively. Flash point, density, and kinematic viscosity of oil were determined to be 94 °C, 874.9 kg/m^3^, and 4.063 cSt, respectively.	Sivagami et al. (2021)

#### Ultrasonic-assisted treatment

Ultrasonic technology is a promising clean methodology for treating oily sludge to recuperate oil. Indeed, several works have been conducted concerning this issue (Gao et al. 2015). In order to improve the ultrasonic treatment protocol, it was investigated how ultrasonic circumstances affected the separation of various oil components from oily sludge. They applied three frequencies (25, 50, and 100 kHz), and the data revealed that ultrasonic treatment at 25 kHz displayed the most effective and efficient rate of oil extraction. Ultrasonic intensity (0.33 W/cm^2^) was fair enough to overcome the energy barrier for oil dewatering. Thus, the optimum conditions for maximum oil recovered were as follows: ultrasonic frequency of 25 kHz, intensity of 0.33 W/cm^2^, and sludge:water ratio of 1:2 (v:v).

In another study, Hu et al. (2016) investigated the effect of ultrasonic assisted solvent extraction process on oil recovery from refinery oily sludge. They investigated the effect of two types of ultrasonic treatment protocols: probe system and bath system. The percentage of oil recovery by these two methods was compared to that of mechanical shaking extraction. Three solvents were used as extraction solvents: cyclohexane (CHX), ethyl acetate (EA), and methyl ethyl ketone (MEK). The effect of several application conditions on oil and solvent recovery was verified using an orthogonal experimental design. The data revealed that the maximum oil recovery was dependent on solvent type, solvent-to-sludge (S/S) ratio, and treatment duration. Under the optimum conditions, oil recovery of 68.8% was obtained within only 20 s. They concluded that this treatment method is promising in recover of oil from the sludge in very short time.

Zhao et al. (2017) investigated the effect of ultrasonic specifications regarding recovering oil from oily sludge, factors including kind of material, bottom thickness, diameter, and supplementary mixing. This operation involved treating oily sludge using purified water and a power of 40,000 Hz at 30 °C in an ultrasonic cleaning tank. The initial oil content of the sludge was 19.29%. Additionally, the wider diameter of the reaction vessel speeds up the evacuation of oil by lowering the ultrasonic wave’s dispersion attenuation.

Although ultrasonic technology is a promising tool for deoiling of oily sludge, little information is available on the pilot scale application of ultrasonic deoiling. Gao et al. (2018) optimized ultrasonic conditions based on the pilot experiment on the effects of ultrasonic properties on the amount of oil separated from an oily sludge. Thus, an ultrasonic power of 0.24 W/cm^2^ and frequency of 25 kHz was applied to overcome the energy barrier for deoiling. Oily sludge content increased from 25.0 to 42.0%, while the clearance rate increased from 46.0 to 60.7%. Additionally, the deoiling process was improved by various ratios when surfactants like sodium petroleum sulfonate and Span 80 were present at an optimized ratio (around 0.3% and 0.03%, respectively). Additionally, a number of pilot scale experiments revealed that using an ultrasonic assistance and surfactants (Span 80), the oil recovery rate was between 82 and 90%.

#### Oil recovery through freezing/thawing

The freezing/thawing has been developed as an alternative method of oil recovery from greasy sludge since crude oil contains various amounts of hydrocarbons according to its origin and locations with variant freezing points of these hydrocarbons. In this method, there are commonly two circumstances in which the separation of the water-phase and the oil-phase occurs. Since the hydrocarbon mixture’s freezing point is lower than that of water in the first scenario, the water phase in the oily sludge will first cool and freeze, expanding in volume. The two phases of the emulsion mixture’s equilibrium will be disturbed by this expansion, and as the temperature is continuously lowered, the oil phase will eventually start to congeal. Crude oil will be separated from the water phase and recovered during the subsequent thawing process as a result of the gravity and surface tension effects on the oil phase (Wang et al. 2015). The second scenario is when the hydrocarbon mixture has a freezing point that is higher than that of water. In this case, the ice that is created by the frozen oil will cover the water phase and the oil phase will begin to freeze before the water phase.

Then, as the temperature continues to drop, the water phase begins to freeze, increasing in volume and forcing the reservoir to overflow. The influence of gravity can be used to separate the oil phase (Hui et al. 2020). Chen and He (2003) investigated the effect of the rate of the freezing process on the dewatering process of an oily sludge, and they discovered that the dehydration impact and oil recovery were both improved by milder freezing and thawing processes. The temperature, time, and rate of freezing and thawing, the quantity of the water phase, oil phase, oxygen, contaminants, and rate of freezing are only a few of the variables that affect how easily oil/water mixtures break up and how easily hydrocarbons may be recovered during the freezing/thawing process.

In order to promote this process, a hybrid of two or more methods can be applied. In this regard, Abdulqawi et al. (2023) combined cyclohexane extraction with freeze/thaw process. The greatest oil recovery rate was achieved with a 4:1 solvent-to-sludge ratio, and a 30-min extraction period was enhanced by 50.98% and about 60.98% of crude oil could be recovered.

In another study, Zhang et al. (2012) combined the method of ultrasound-freeze/thaw treatment to treat a tank bottom sludge and the three-phase separation mechanism was discussed. The optimal conditions were as follows: the ultrasonic frequency was 28 kHz, temperature was 5 °C, ultrasonic power was 350 W, ultrasonic time is 60 min, the freeze temperature is −5 °C, the freezing time is 3 h, and the thawing temperature is 60 °C. At these conditions, about 65% of oil was recovered. Indeed, there are other treatment protocols are being applied with lesser progress. These are incineration, oxidation, bioremediation, and solidification. Each of these methods are only applied under certain requirements such as low quantity of sludge and high-temperature environment, and suitable conditions. A comparison between the most commonly applied methods with their cons and pros is held in Table 5.

**Table 5:** Cons and pros of the most common sludge treatment protocols

The applied protocol	Advantages	Disadvantages
Solvent extraction	Cost-effective, variability of solvents, can be combined with other methods, low application requirements	Low efficiency and unpredictability. Sometime environmentally prohibited
Chemical demulsification	Availability of green demulsifiers (Adewunmi et al. 2020; Yang et al. 2022b), biosurfactants (Chirwa et al. 2013; Liu et al. 2018), diversity of different surfactants	Involves multistage operations, require high concentration of surfactants, usually combined with thermal or mechanical treatments (Duan et al. 2019)
Pyrolysis	Lack of costly separation processes; ability to process raw materials with high ash contents, including agrowastes; avoidance of consumables (like solvents); absence of waste and items that need to be disposed of (Petrovsky et al. 2019)	Uncontrolled, high temperature process, production of several byproducts and undesired mineral materials (Petrovsky et al. 2018)
Freezing/thawing	Can promptly be applied in regions of cold weather (Jean et al. 2001)	More restrictive, more costly and less economical (Wang et al. 2017a)

#### Reduction of contaminants

The usual routine practice of sludge after recovering valuable hydrocarbons is the disposal or landfilling of sludge which cause enormous air and soil pollution as some greenhouse gases such as methane and CO_2_ are produced during sludge bioremediation. Additionally, petroleum sludge contains contaminants such as heavy metals, dissolved solids, and sulfur compounds that may pose environmental risks (Kondaveeti et al. 2023). The common methods applied for sludge disposal are incineration, oxidation, solidification/stabilization (S/S), and biodegradation (Table 6). However, safe, fast, and effective regimes must be adapted in order to recover most of the materials comprising the sludge and to ensure economic and cost-effective values (Hamidi et al. 2021).

**Table 6:** Common sludge disposal protocols

Methodology	Description	Remarks	Ref.
Incineration	Waste sludge is completely burned outside with additional fuel. The rotary kiln and fluidized bed incinerator types were the two most commonly used. While combustion temperatures in fluidized beds varied from 730 to 760 °C and required days to complete, combustion temperatures in rotary kiln incinerators ranged from 980 to 1200 °C and required only 30 min.	Sludge with a high-water content needs to be pre-treated to lessen its viscosity before being charged into the incinerator. Due to its high mixing efficiency, fuel variety, low pollutant productions, and high combustion efficiency, fluidized beds are used in the treatment of low-quality sludge.	Scala and Chirone (2004)
Stabilization/solidification	It includes encasing or sealing waste with an adhesive in order to stop the waste from leaking into the surrounding environment.	Changes to the chemical pollutants are made through chemical overlapping between the cement's products of hydration and the impurities themselves.	EPA (1993); Zain et al. (2010)
Oxidation treatment	Utilizing chemical and other oxidation agents, oxidation treatment is the process of degrading organic pollutants found in the disposal outcome of sludge.	When the oily sludge is exposed to the reactive agents, organic substances will oxidize to produce carbon dioxide and water or non-hazardous materials. Fenton’s reagent, hypochlorite, ozone, ultrasonic irradiation, permanganate, and persulfate are a few examples of the several oxidation reagents that can be used.	Cui et al. (2009); Ferrarese et al. (2008)
Bioremediation	In land treatment, bio pile/composting, and biological-slurry, microorganisms have been used to biodegrade and remove environmental toxins.	Time-consuming and less efficient	Wang et al. (2012)

In this regard, Bhattacharya et al. (2021) conducted an important study aiming to establish an appropriate benign sustainable methodology for the cleanup of petroleum industry-produced oily sludge. The remediation of oily sludge was examined in this study using three experimental sets, including a control set (C-set), a set with compost (C+ set), and a set with silver (Ag) nanoparticles (N set), under various application conditions, including pH, conductivity, heavy metals (Pb, Ni, As, and Fe), and total petroleum hydrocarbon (TPH). They discovered that the C+ set had the highest removal rates for the following heavy metals: Fe (91.77%), Pb (54.44%), Ni (76.11%), As (33.48%), and TPH (65.52%). The correlations between pH and TPH, as well as between As (arsenic) and Fe (iron), were all positive, while the correlations between conductivity and TPH were all negative. The prospective ecological risk index showed low levels of danger for Pb (0.5–0.30) and Ni (0.043–0.064), whereas As (67) in the N set and C− (88) and C + (107) set showed moderate levels of risk and higher levels of risk, respectively.

Aitkaliyeva et al. (2022) solved the issue of sludge disposal by processing the sludge produced from different oil industries into valuable construction and road materials and investigated the possibility of using different sludges as an additive for bitumen grade BND 70/100.

Xiao et al. (2019) used the solidification/stabilization (S/S) approach to mix oily sludge as the main component, fly ash, silica fume, commercial cement made from Portland cement as binders, and phosphogypsum (PG) as a stabilizer. Various tests, such as an unconfined compressive strength test and a toxicity leaching test, were used to determine the operation’s efficiency. It was discovered that adding 20% binders and phosphogypsum to oily sludge improved the samples’ 28-day compressive strength and dramatically decreased the release of heavy metals while also improving the pore structure and compacting the microstructure.

Another work conducted by Adigwe et al. (2022) concerned the employment to eliminate environmental concerns brought on by the dumping of untreated sludge; microbiological analysis is a bioremediation alternative for remediating petroleum sludge. The samples of grown sludge yielded a number of strains. Following screening, Aspergillus flavus, *Aspergillus niger*, *Verticillus* sp, *Penicillum* sp, and *Microsporium audouinii* were the organisms’ nominated strains.

The reduction of the total hydrocarbon content (THC) and the percentage composition of the THC in the five reactors after treatments were assessed using the usual Friedman non-parametric test after 12 weeks of treatment. The THC in the treated sludge decreased by anywhere between 56.0 and 67.3%. These demonstrated the potential for improved biodegradation of petroleum sludge by microorganisms that use hydrocarbons.

The percentage of oil recovery by some methods is given in Table 7.

**Table 7:** The percentage of oil recovery of some methods applied in lab or field

The method applied	Scale	% of oil recovery
Surfactant (EOR)	Field	80
Freeze/thaw	Laboratory	59
Pyrolysis	Field	68
Microwave irradiation	Field	89
Electro- kinetic	Laboratory	92
Ultrasonic	Laboratory	78
Incineration	Field	92
Oxidation	Laboratory	90
Biopile/compositing	Field	82

## Future perspectives of treatment protocols

The analysis and evaluation of data included in this review indicated that there is a consistent rise in studies on oily sludge which reflects the significant importance of this issue. Different points can be pointed out to enhance the processes applied for handling of TBS; these are the following:

- It is advised that greater funding from the petroleum industry sectors be provided to aid in the researches concerning the problematic issues in petroleum sector. It is important to apply the research findings from small-scale trials conducted in a lab on field scale operations and to verify the cost-effectiveness of the proposed treatment methods. Funding is also needed to cover the expenses paid for the materials adopted and to simulate the actual operation conditions. For real applications, the results of the research should be advanced from laboratory-scale tests utilizing materials that are easily accessible and reasonably priced. This will allow an exact estimation for the overall process and facilitate a reasonable cost-benefit analysis of a proposed technique or approach for handling oily sludge.
- In spite of the variation of the methods that have been developed and applied, only few of them could accomplish satisfactory therapeutic results when utilized. Therefore, combination of two or more methods is highly desirable. This emerges the requirement for an integrated strategy while developing oily sludge treatment methods.
- Approaches such as sustainability and circular economy must be kept in mind when developing, applying and establishing new research work on sludge treatment. Moreover, valorization of the materials recovered from the sludge requires more studies. Therefore, more efforts must be pushed to create generally recognized assessment methods for the assessment of oily sludge’s environmental danger when selecting of suitable methods for the treatment of oily sludge.
- Also, the factors affecting the formation of the sludge must be carefully studied in order to reduce the amount of the sludge generated at each step of operations.
- Although some treatments are incredibly promising for fuel recovery and/or decontaminating unrecoverable wastes, but their large-scale implementation alternatively they might have very high operational expenses, restricting their feasibility. This point must be addressed.
- Updated characterization techniques must be applied to select the treatment technologies that exactly meet the needs of sludge characteristics, treatment capacity, prices, legal and governmental disposal regulations, and time restrictions.

Finally, the most effective treatment strategy can be reached by incorporating many approaches into a process train. For instance, to achieve remarkable oil recovery performance, the ultrasonic irradiation method can be used with the freeze/thaw process to treat oily sludge. (Teng et al. 2021). Fenton’s oxidation and solidification/stabilization techniques can be combined to offer an environmentally benign oily sludge disposal (Jagaba et al. 2022) and so on.

## Conclusions

Oil sludge is a hazardous material produced through different operations in petroleum industry. Tank bottom sludge is of particular importance because it is considered a valuable fuel material. In addition, the illegal disposal of this sludge may induce several environmental effects and leads to health risks beside huge economic loss. Sludge formation, composition, hazardous effects, and the most important methods applied to recover oil or to recycle tank bottom sludge has been extensively reviewed. Some of these methods include solvent extraction addition of surfactants, freezing thawing, and incineration. The advantageous and disadvantages of each method were also mentioned. The benefits of combining two or more methods were also stated.

## Data Availability

This work contains the data that back up the study’s conclusions.

## Declarations

### Acceptance of participation

The authors reaffirm that all of the data and findings from the experiments they used in this work are accurate.

### Permission to publish

The authors agree to the publication of personally identifiable information in the aforementioned journal.

## Abbreviations

MEK: methyl ethyl ketone
LPGC: liquefied petroleum gas condensate
PACs: polyaromatic hydrocarbons
LPG: light petroleum gases
API: American Petroleum Institute
FFU: flocculation–flotation unit
IAF: induced air flotation
mboe/d: million barrels of oil equivalent per day
VOCs: volatile organic compounds
PHCs: petroleum hydrocarbons
EC: electrical conductivity
TSD: total solid, density
COD: chemical oxygen demand
TOC: total organic carbon
CPI: corrugated plate interceptor
DAF: dissolved air flotation
PHCs: petroleum hydrocarbons
BTEX: benzene, toluene, ethylbenzene, and xylene
SARA: saturates, aromatics, resins, and asphaltenes
